# Nasotracheal intubation-extubation-intubation and asleep-awake-asleep anesthesia technique for deep brain stimulation

**DOI:** 10.1186/s12871-019-0685-y

**Published:** 2019-01-17

**Authors:** Wenxi Tang, Penghui Wei, Jiapeng Huang, Na Zhang, Haipeng Zhou, Jinfeng Zhou, Qiang Zheng, Jianjun Li, Zhigang Wang

**Affiliations:** 1 Department of Anesthesiology, Qilu Hospital of Shandong University (Qingdao), No.758 Hefei Road, Qingdao, People’s Republic of China; 2 Department of Anesthesia, Jewish Hospital and Department of Anesthesiology & Perioperative Medicine, University of Louisville, Louisville, KY USA; 3 Department of Neurosurgery, Qilu Hospital of Shandong University (Qingdao), Qingdao, People’s Republic of China

**Keywords:** Asleep-awake-asleep, Deep brain stimulation, Nasotracheal intubation, Parkinson’s disease

## Abstract

**Background:**

The asleep-awake-asleep (AAA) technique and laryngeal mask airway (LMA) is a common general anesthesia technique for deep brain stimulation (DBS) surgery. However, the LMA is not always the ideal artificial airway. In this report, we presented our experiences with nasotracheal intubation-extubation-intubation (IEI) and AAA techniques in DBS surgery for Parkinson’s disease (PD) patients to meet the needs of surgery and ensure patients’ safety and comfort.

**Case presentation:**

Three PD patients scheduled for DBS surgery had to receive general anesthesia for various reasons. For the first asleep stage, general anesthesia and nasotracheal intubation was completed with routine methods. During the awake stage, we pulled the nasotracheal tube back right above the epiglottis under fiberoptic bronchoscope (FOB) guidance for microelectrode recording (MER), macrostimulation testing and verbal communication. Once monitoring is completed, we induced anesthesia with rapid sequence induction and utilized the FOB to advance the nasotracheal tube into the trachea again. To minimize airway irritations during the process, we sprayed the airway with lidocaine before any manipulation. The neurophysiologists completed neuromoinitroing successfully and all three patients were satisfied with the anesthesia provided at follow-up.

**Conclusion:**

Nasotracheal IEI and AAA anesthetic techniques should be considered as a viable option during DBS surgery.

## Background

Monitored Anesthesia Care (MAC) is the most popular anesthesia technique in DBS surgery for PD patients. However, general anesthesia might be the only option for some patients. The major challenge with DBS under general anesthesia is the requirement of a fully awake and communicative patient for microelectrode recording and macrostimulation testing. The Asleep-Awake-Asleep (AAA) technique may be suitable in this situation. There have been multiple reports of this technique with laryngeal mask airway (LMA) for other neurosurgical surgeries [1]. However, LMA does not always guarantee a secure airway [2]. In this report, we presented our experiences with nasotracheal IEI and AAA techniques in PD patients to meet the needs of surgery and ensure patient safety and comfort.

## Case presentation

The first patient was a 63-yr-old female with Body Mass Index (BMI) 19.8 kg/m^2^. She presented to our hospital for DBS implantation. Unfortunately, she suffered severe kyphosis and could not tolerate supine position. The second patient was a 56-yr-old male with BMI 24.4 kg/m^2^ suffering from severe back pain and anxiety. Both patients refused MAC for surgery. The third patient was a 64-yr-old male and the BMI was 28.7 kg/m^2^. This patient had severely uncontrollable motor symptoms and Obstructive Sleep Apnea syndrome (OSA) (Apnea Hypopnea Index is 33). His OSAS was triggered and the head movements associated with snoring also hampered the preoperative MRI scan when we gave dexmedetomidine to reduce the body movement. Only after the OSAS was eliminated by placing a nasopharyngeal airway to overcome the upper airway obstruction, the MRI scan was finished successfully. This made MAC is a poor choice for his DBS surgery. All three patients agreed with our proposed IEI and AAA technique. Written consent from patients and Institutional Review Board approval were obtained. During preoperative interview, we described the protocol of arousing, extubation, macrostimulation testing and reintubation in great details. On the day of surgery, The Leksell stereotactic head frame was placed under local anesthesia before entering the operation room.

### The first asleep stage

**Fig. 1:**
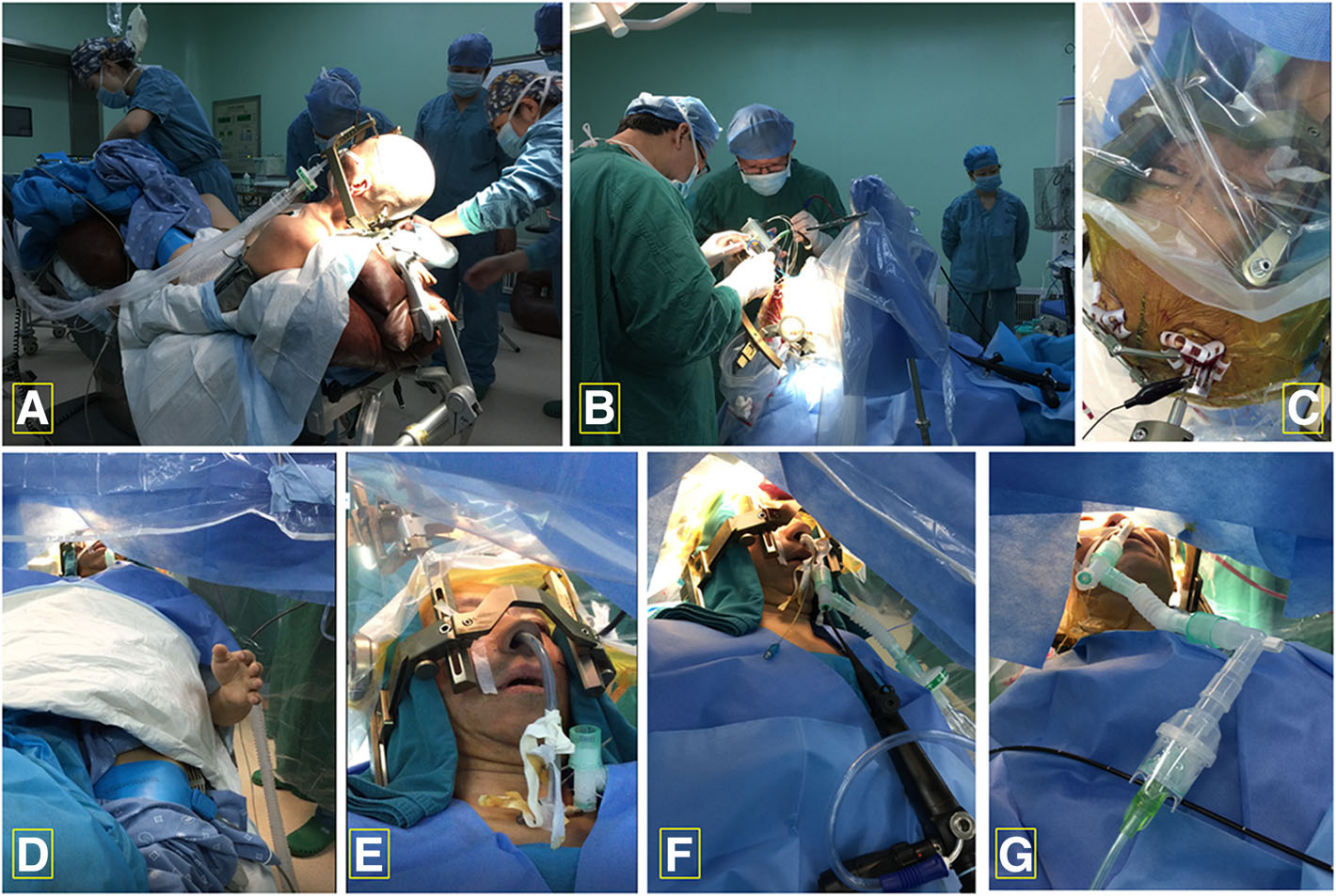
**a** The severe hunchback of the first patient and methods to keep the patient in the supine position, **b** The other two patients were in routine supine position. **c**-**e** During macrostimulation testing, patients were calm and cooperative, able to move fingers, limbs, and to communicate verbally with the operator. **f** The reintubations guided by FOB. **g** Inhaling atomized 2% lidocaine via nasotracheal tube with catheter mount and a simple atomizer

After entering the operation room, dexmedetomidine 0.4μg/kg was given within 15 min. We kept the first patient in the supine position with multiple cushions (Fig. 1, Part A). The other two patients were placed in routine supine position (Fig. 1, Part B). The oxygen saturation, expired carbon dioxide, ECG and invasive blood pressure were monitored. One nostril was sprayed with 1% ephedrine, then the nasal and oral mucosa were anesthetized with 1% dyclonine gel. After glycopyrrolate 0.2 mg and palonosetron 0.25 mg i.v., general anesthesia was induced with dexamethasone 5 mg, fentanyl 2μg/kg, propofol 1-2 mg/kg and atracurium 1 mg/kg. After 3 min of mask ventilation, the laryngeal and tracheal mucosa was anesthetized with 2% lidocaine (5 ml) through a disposable endolaryngeal anesthetic tube sprayer (Henan Tuoren Medical Device Co., Ltd., China) under video laryngoscope guidance (Aircraft Medical Ltd., Ediburgh, United Kindom). A nasotracheal tube (ID 6.5 for male and ID 6.0 for female) was advanced past the vocal cords and the distance between the nare and the epiglottis was recorded. General anesthesia was maintained with 4 mg/kg/h propofol, remifentanil 0.1μg/kg/min and dexmedetomidine 0.2μg/kg/h. Local anesthesia to operative sites was provided with 0.325% ropivacaine by surgeons.

### The awake stage

Ten minutes before the anticipated microelectrode recording (MER), dexmedetomidine, propofol and remifentanil were discontinued. After patient’s spontaneous respiration was restored, we deflated the cuff of endotracheal tube and injected atomized 2% lidocaine 3 ml via a catheter mount and a simple atomizer (Jiangsu Sona Care Medical Science-Technology Co., LTD, Nantong, China) (Fig. 1, Part G). The endotracheal tube was retracted to the top of epiglottis (at level of the tongue base) under the guidance of FB-10 V FOB (HOYA Corporation, PENTAX Lifecare Division, Tokyo, Japan) and kept there as a nasopharyngeal airway. When the patient woke up fully, MER, macrostimulation testing and language communication were performed. All three patients opened their eyes upon commands (7 ± 0.66 min) and their spontaneous respiration restored within 5–10 min after stopping sedation. They all tolerated nasal endotracheal tube well (Fig. 1, Part C). Both blood pressure and heart rate were significantly higher than asleep stage. Compared with baseline blood pressure, the fluctuation of mean arterial pressure (MAP) of the first patient and third patient were within 30% and we did nto give any treatment. The MAP of the second patient was higher than 30% baseline blood pressure, we gave nicardipine (0.1 mg) and esmolol (0.5μg/Kg) intermittently to maintain hemodynamic stability (the total dose of nicardipine was 0.3 mg, esmolol was 100μg).

The neurophysiologists finished MER successfully and were satisfied with the quality of signals. During the macrostimulation testing, all the patients were calm and cooperative, able to move fingers, limbs, to count numbers upon instructions and to communicate orally with the operator easily (Fig. 1, Part C, D, E).

### The second asleep stage

Once the electrophysiological test was complete, atomized 2% lidocaine 3 ml via nasotracheal tube was injected again. Midazolam 0.04 mg/kg, fentanyl 2μg/kg, propofol 1 mg/kg were utilized to sedate patients and maintained spontaneous respirations. The glottis was identified with FOB and oxygen was supplemented through the catheter mount. Once the FOB entered the trachea, the nasotracheal tube was passed over the FOB into the trachea (Fig. 1, Part F). Rocuronium 1 mg/kg, remifentanil 0.5μg/kg and propofol 1 mg/kg were injected intravenously to induce general anesthesia. The rest of the procedure, such as implantation of electrodes and pacemakers, was continued under general anesthesia. The reintubation guided by FOB were successful on the first attempt in all three patients (Fig. 1, Part F).

Total AAA time were 235–280 min, including first asleep stage 80–100 min, wake-up test time of 58–70 min, and the second asleep stage 97–110 min. No patient had coughing or body movement. One patient suffered mild nose bleeding but had no significant impact on nasotracheal reintubation guided by FOB. No patient suffered hypoxia during the whole process. All patients were extubated within 10mins after operation without complication. Anesthesia follow up on postoperative day 2 demonstrated that all patients were satisfied with their anesthesia experiences.

## Discussion and conclusions

We did not find reports on nasotracheal IEI and AAA technique after Huncke K, et al. published their technique for intractable seizure resection in 1998 [3]. Our technique has several noticeable improvements compared to Huncke’s method. First, we did not remove nasotracheal tube during awake stage. Instead, we kept the nasotracheal tube above the epiglottis in the nose, which could keep airway unobstructed. This could prevent additional trauma and bleeding from placing another nasopharyngeal airway in patients with airway obstructions. In addition, this technique will not impair the necessary oral communication testing and could make reintubation easier by reducing the difficulty to find the glottis with FOB. Second, both intubation and reintubation by Huncke K were performed while patients were awake. For the first asleep stage, intubation by us was performed after induction of general anesthesia. For the second stage, we identified the glottis and intubated trachea using FOB when the patient was heavily sedated. Patient safety and comfort during the intubation are improved and the difficulty of anesthesia management is reduced. High-flow nasal oxygen therapy or high frequency ventilation during the apnea period might be useful to prevent hypoxia [4]. Third, Huncke K, et al. used a spirally attached catheter around the endotracheal tube to provide airway anesthesia, we used a disposable endolaryngeal anesthetic tube spray before the first intubation, a simple atomizer before the first extubation and second intubation. Endotracheal surface anesthesia before intubation is the most important factor to make patient tolerable to intubation or extubation. Fourth, Huncke K used propofol, sulfentanyl or inhaled agents and they needed to be discontinued at least 1 h before the wake-up. We maintained general anesthesia with short acting propofol, remifentanil and low-dose dexmedetomidine. Only 10mins was required before the wake-up. All of our patients were able to wake up quickly and cooperated with neurological tests. Dexmedetomidine could reduce anxiety, airway sensitivity and increase analgesic effects but had no significant impact on MER monitoring [5, 6]. Finally, DBS was performed in the supine position and the airway was easily accessible to the anesthesiologist. In other neurosurgical cases, it may require extra personnel or devices to tent the surgical drape to facilitate airway management as reported by Huncke K.

Possible advantages of our IEI technique in comparison with LMA techniques are: First, the supra-epiglottis position of the endotracheal tube could serve as a nasopharyngeal airway and reduce the risk of hypoxemia. Second, the depth of anesthesia required is lower in IEI and can be performed when patients are conscious. Third, the successful rate for a secure airway with video laryngoscopy may be higher than that of LMA when the head position is fixed. Finally, this technique can potentially prevent aspiration with a secured airway. Potential disadvantages include: First, the possibility of significant nasal bleeding causing laryngeal irritation. Second, airway swelling from repeated instrumentations. Third, advanced skills to manipulate the FOB are required.

Our experiences suggest that nasotracheal IEI and AAA techniques should be considered as a viable option for some certain patients undergoing DBS surgery. Compared with local anesthesia alone, this technique could shorten the awake time and improved patient’s satisfaction. In addition, the secured airway with an endotracheal tube may be safer than LMA. The advantages and disadvantages of this technique should be verified by more rigorous research.

## Abbreviations

AAA: Asleep-awake-asleep
DBS: Deep brain stimulation
FOB: Fiberoptic bronchoscope
IEI: Intubation-extubation-intubation
LMA: Laryngeal mask airway
MAC: Monitored Care Anesthesia
MER: Microelectrode recording
PD: Parkinson’s disease
